# The Shakespeare Myth

**Published:** 1912-12

**Authors:** V. Edwin Durning Lawrence

**Affiliations:** 13 Carlton House Terrace, London, England


### The Shakespeare Myth

To the Editor MEDICAL JOURNAL, Buffalo:

Sir.—As in your October issue you referred to the above, I think you would be doing a great service to the English speaking race everywhere, if you would accord us a little of the valuable space in your journal which has so wide a circulation.

When Bacon was born the English tongue, as we now understand it did not exist. And when as a youth he saw in Paris what the Pleade (the seven men) had done in France by changing what they described as a barbarous jargon into an elegant French language, Bacon determined that he would do as much for England and create an English language capable of expressing the highest thoughts.

At that period very few books existed in the English tongue that could be considered to be literature, excepting "The Schoolmaster," by Roger Ascham, "The Governor," by Sir John Eliot, and "Arts of Rhetoric," by Thomas Wright.

In 1566 a catalogue was made of Sir Thomas Smith's library of about 1,000 volumes. Only five of these were in English, viz: Henry VIII, Theology, Literature, Tenures (law); Hill, Chronicles; Fabian, Chemistry, etc., and Art of Navigation, all these being technical works.

A grammar school was a Latin grammar school where English was not taught and where Latin was never translated into English.

In 1580 when Bacon was 20 years old the Statutes of Harrow School provided that no boy excepting in the lowest form should speak anything but Latin even in the playground.

I desire to impress upon your readers the fact that our present English language was created by Francis Bacon, and that there was not a single writer of any importance in "the Elizabethan golden age of literature" who wrote independently of Bacon's instructions.

All writers are agreed that the English language of today is founded upon the 1611 English translation of the Bible and upon Shakespeare's plays.

In the preface of the Bible of 1611 we are told that great pains had been taken to ensure that no good English word should be excluded; and J. A. Weisse tells us that about 15,000 different words are employed in the Bible. With respect to Shakespeare (we mean the folio of 1623), Max Müller tells us it contains 15,000 different words. This is, however, an under-estimate. Among the 2,000,000 words contained in the plays there are about 22,000 different words, 7,000 of which are new words which, as Murray's New Oxford Dictionary tells us, are introduced into the English language for the first time by Shakespeare. Does any one suppose that the illiterate Stratford clown knew more than 300 or 400 words? Although the English language was thus enormously enriched by Shakespeare, and although many thousands of words have been added since his time, yet neither Dickens nor Thackery made use of more than 7,000 or 8,000

different words in all their writing. Max Müller informs us that "a well-educated person who has been to a public school and an university * * seldom uses more than 3,000 or 4,000 words."

In the preface to the Bible we are also told that great endeavors had been made to secure a variety of expression, and Max Müller states in reference to Shakespeare that he "probably exhibits a greater variety of expression than any writer in any language."

About 48 persons whose names are known translated the Bible for the 1611 version. But, as Archdeacon Westcott, writing in the *Times* of March 22, 1912, says in reference to the 1881 revised version of the Bible, "successful literary results cannot be achieved by syndicates. Anyone turning over the pages of the Authorized Version of the Bible, and reading half-a-dozen verses here and half-a-dozen verses there in the books that compose the volume (aloud by preference) cannot fail to perceive that the grand rhythm which runs throughout the whole is not the work of a syndicate, but must be the product of one great master mind.

The 1611 Bible is ornamented with Baconian symbols, and in my own special copy of the second edition (also 1611) these symbols are Rosicrucianly marked to call the attention of the initiated to them, and to tell them that the 1611 Bible is without possibility of doubt one of Bacon's books.

Bacon's secrets have always been known to certain leading Free Masons, whose number was strictly limited to nine, until 1910. But in that year, which is 287 years after the issue of the folio of the "Shakespeare" plays in 1623, the number of fully "informed" Masons was raised from nine to thirty-three. Outside of that "circle" I do not suppose there are more than three or four persons besides myself who are "informed" of this fact.

When Bacon was born English as a literary language did not exist, but ere he died he had succeeded in making the English language the noblest vehicle of thought ever possessed by mankind. This he accomplished mainly by *his* BIBLE and *his* SHAKESPEARE.

V. EDWIN DURNING LAWRENCE,
13 Carlton House Terrace, London, England.

Note. We are glad to print the above interesting letter, in spite of its being rather outside of the scope of the JOURNAL. While recognizing the great influence of Bacon in perfecting the English language, it must be conceded that it was a good deal more than a jargon and had a considerable literature before his time. English, as a Franco-Saxon development distinct from Anglo-Saxon, probably began to be formed even before the Conquest of 1066 for the Normans had obtained a foot-hold in England before William. The English language supplanted French in courts of law in 1362 and, at about this time, Chaucer and others wrote fairly good literature in English that is quaint but but still understandable. As we stated in the review of Mr. Lawrence's book, anyone competent to judge, contemporaneously or otherwise, would have suggested Bacon as the most probable author of "Shakespeare" if the works known by that name had been anonymous. But it does not seem probable that an "illiterate Stratford clown" could have had Shakespeare's forehead, enjoyed the local prestige, or mingled with London society, as did Shakespeare, if he had been merely a tool. It is not so far outside of medical interests to declare boldly that Max Müller's estimates of vocabulary are ridiculously low.

